# Using the fuzzy logic approach to extract the index of economic sanctions in the Islamic Republic of Iran

**DOI:** 10.1016/j.mex.2021.101301

**Published:** 2021-03-10

**Authors:** Saeed Iranmanesh, Norallah Salehi, Seyyed abdolmajid Jalaee

**Affiliations:** Department of Economics, Faculty of Management and Economics, Shahid Bahonar University of Kerman, Kerman, Iran

**Keywords:** Index of economic sanctions, US sanctions, UN Security Council sanctions, EU sanctions, Fuzzy logic

## Abstract

Fuzzy logic is one of the most widely used methods for quantifying qualitative studies. Because based on the fuzzy logic method, a wide range of verbal interfaces can be presented quantitatively. Fuzzy logic is a form of multi-valued logic. Quantitative indicators for qualitative events can be defined with its help. Iran has faced the phenomenon of economic sanctions since 1979 and after the victory of the Islamic Revolution. These sanctions have had different types and have affected different sectors of the Iranian economy. However, in most cases, studies on these sanctions have been limited and inefficient. In this study, based on the fuzzy method, an attempt has been made to define a quantitative index for economic sanctions during the years 1979–2019, using the opinions of economics experts on sanctions. This quantitative index can be utilized as a useful time series. Researchers in the field of Iranian economics and the economics of sanctions can help to study the impact of this economic phenomenon on different sectors of the Iranian economy. The results of this study showed the index of sanctions during 2011 and 2012, in which the most sanctions were imposed against Iran. The index was extracted and it was shown that it had the highest value.

• In qualitative studies, to quantify the studied trends, the method of fuzzy logic and conversion of verbal propositions into numbers and figures can be used.
• The index of economic sanctions is a useful time series for researchers in the field of sanction economics and the Iranian economy.
• The index of economic sanctions can determine how each of the economic sanctions against Iran will work.

## Graphical abstract

**Figure:**
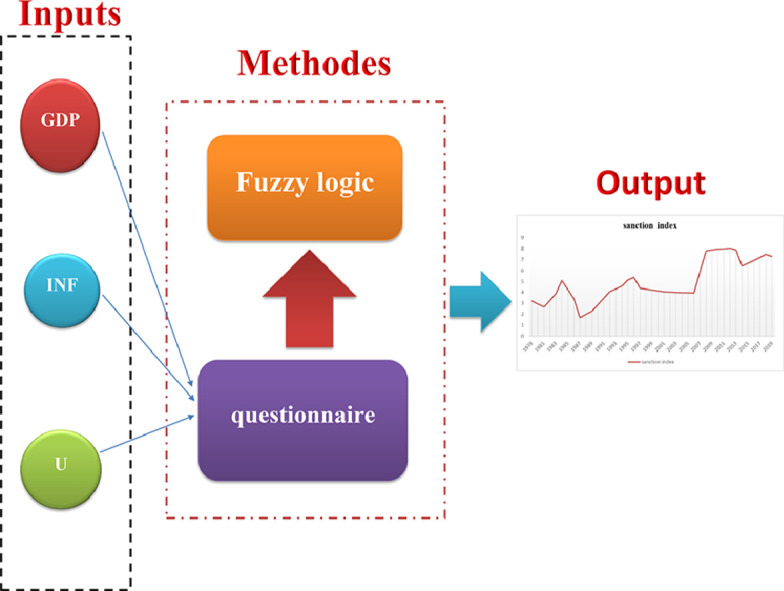

Specifications table

**Table:** 

Subject area:	Economics and Finance
More specific subject area:	Macroeconomics - International economics
Method name:	Fuzzy logic
Name and reference of the original method:	**Fuzzy logic**: Zadeh, L. A. (1776). Toward Human Level Machine Intelligence—Is It Achievable? The Need for a Paradigm Shift. University of California, Berkeley, 94720.‏
Resource availability:	**Data source location:**https://info.worldbank.org/governance/wgi/ https://www.heritage.org/index/explore?view=by-region-country-year&u=637188530188497349 https://databank.worldbank.org/reports.aspx?source=world-development-indicators# http://www.hdr.undp.org/en/data# questionnaire **Software:** MATLAB

## Introduction

Nowadays, fuzzy methods alone and in combination with other methods are widely used in various studies. Due to the special and wide features of the studied topics, currently there is a greater need to use accurate methods. Qualitative phenomena need to be accurately evaluated from different aspects. In addition, the indicators introduced to study qualitative phenomena must be comprehensive enough and carefully evaluate all the aspects affecting a phenomenon.

Iran has always been concerned with economic and political developments in the Middle East. The special capabilities and advantages of Iran in various fields, such as access to energy, significant size, and population, abundance and diversity of mineral resources, and strategic location, play a significant role in this country. By being present in the Middle East, understanding and using its situation and advantages, while creating new opportunities, Iran can highlight its role in the region and in addition to helping in the development of the country, be fruitful in achieving peace and developing the region [1].

The United States and its allies have been confronting Iran to halt its nuclear program. They have used four different political tools to force Iran to abandon its nuclear program: Economic sanctions, media propaganda, the threat of military action, and diplomacy [2]. This behavior change can be due to a change in political behavior or a change in economic policies. Using economic, financial, and technological tools, one of the effective ways to implement foreign policy is to achieve security goals and interests [3].

Sanctions are a punitive tool used in foreign policy. The purpose of sanctions is to pressure the target country to change its behavior [3]. Among the different types of sanctions, economic sanctions are the most widely used in the world. There are various types of economic sanctions, including export tariffs, import quotas, trade bans, and non-tariff barriers to imports [4].

So far, the fuzzy logic method has been used to index various natural and social phenomena. In his study, Raman examined the water quality index and the importance of water quality parameters using the fuzzy logic method. He extracted water quality indicators by forming a fuzzy network based on the input and the output [5]. In the study by Gentile, the fuzzy logic method was used to calculate the intrinsic safety index [6]. Moreover, Hendiani in his study relied on the fuzzy method to extract the index of social stability of fuzzy construction [7].

Extensive studies have been conducted on economic sanctions. Shahrestani states in his study that the Iranian economy experienced various types of sanctions after the revolution and during the war with Iraq. The sanctions have led to rising inflation, gasoline quotas, declining non-oil exports, and declining foreign direct investments. The main difference between the current sanctions imposed on Iran and the sanctions imposed during the war is that the recent series of sanctions are supported by the international community in a way that puts more pressure on Iran's economy and ties the hands of policymakers, encouraging them to react more accurately. However, Iranian officials believe that since economic sanctions have been imposed on Iran in the past and the country has overcome these problems, the negative consequences of the new measures can be minimized. For example, they argue that sanctions have increased the country's self-sufficiency and led to a redistribution of resources in development projects. However, specific opportunity costs are associated with these seemingly positive aspects. These sanctions affect the Iranian economy through various channels of the transfer mechanism. The most important things that the authors emphasize in this article are inflation expectations, exchange rate fluctuations, additional financing costs, real estate prices, foreign direct investment, total factor productivity, and economic growth [8].

Moreover, Ismail in his study aimed to analyze the determinants of terrorism in Pakistan. The determinants of terrorism include various socio-economic variables such as per capita gross domestic product (GDP), unemployment, political rights, inflation, poverty, inequality, and literacy levels. A long-run relationship between variables was analyzed using Johansen's common integration method. The error correction model (ECM) was used to determine the stability of the long-run relationship between terrorism and various variables, as well as to simplify the short-term and long-term effects of variables on terrorism. Overall, the results showed that there is a long-term relationship between different socio-economic variables and terrorism, while the ECM results showed that each year about 89% converge towards equilibrium. Similarly, important results were obtained with short-term and long-term elasticity estimated in the ECM. Impact response analysis showed that the effects of a standard deviation shock given to random disturbances on the system of variables have different results. Some variables have an increasing trend over time, some have a decreasing trend, while others have oscillating and periodic trends [9].

Shearkhani, in a study entitled ``Study of the effectiveness of sanctions on Iran's non-oil trade (model of gravity)'', while reviewing the historical background of sanctions against Iran, investigated the direct effect of economic sanctions on Iran's non-oil trade and evaluated a sample of 42 business partners of the country between 1977 and 2006 using the model of gravity. To investigate the effect of sanctions, this study used both broad and medium sanctions as two virtual variables affecting the slope coefficient. The results showed that extensive and medium-sized sanctions, although politically ineffective on Iran's work process, had statistically significant effects on Iran's exports and imports with its trading partners up to that point [10].

The present article examines the economic sanctions imposed on Iran by the United States, the United Nations Security Council, and the European Union.

There are different views on the impact of economic pressures. Careful statistical studies are needed to measure the impact of these policies. Iran has attracted the attention of many Western countries due to its significant growth in recent years and has always been subject to various political and economic sanctions and witnessed special and unique economic conditions, some of which have never been seen in the world. Therefore, when special conditions arise without a pattern, there is a need for innovation and ideation of experts to meet economic challenges.

The purpose of this article was to extract the data related to the index of economic sanctions against the Islamic Republic of Iran. One of the data needed in the current situation in Iran is the data that can determine the impact of economic sanctions on the Iranian economy. To have a wide range of opinions, the fuzzy logic method was chosen as a verbal numerical interface. Sanctions imposed on Iran since 1979 have affected various sectors of the economy. Therefore, first, the questionnaires were prepared based on the analysis points in the main macroeconomic variables. In these questionnaires, each individual's opinions about the impact of each of the sanctions imposed on the Islamic Republic of Iran on the variables of GDP, unemployment rate, and inflation rate are collected. The reason for using these variables to derive the index of economic sanctions is that in most studies on the effect of sanctions on Iran's economy, it has been attempted to examine the effect of sanctions on economic growth, inflation, and employment. Moreover, these variables are considered to be basic macroeconomic variables [8], [9], [10]. In addition, by consulting with research experts, these variables were selected as the main variables used to extract the index of sanctions. The views of 15 activists and economists on the sanctions imposed on Iran were collected. The participants had a doctorate in international economics and a master's degree. Then, using the fuzzy information collected from the experts and processing this information through fuzzy logic in the MATLAB software space, a series of sanction indexes for the Iranian economy were extracted. Following the introduction, this article reviews the studies that have so far been conducted on sanctions in the field of economics. Then the theoretical foundations of indexing and fuzzy logic are discussed and the results of fuzzy logic are extracted. In the end, according to the time series of the sanction index, the conclusion is presented.

## Method details

### Theoretical foundations

Presently, the need for sophisticated techniques and the application of precise research methodology is increasingly felt. In terms of the specific quality of the attributes and variables that should be measured and evaluated, and due to the higher quality of these attributes, it is necessary that the indicator used to measure these attributes depicts various aspects of the concept and has the necessary comprehensiveness. To measure abstract concepts, it is essential to understand their different aspects by asking different questions. However, to quantify the concept in question, so that it is possible to measure each unit of the sample and finally a general conclusion is feasible, it is necessary to combine and integrate different components for each concept. This is called ``index construction''.

A quantitative index represents several homogeneous variables and is a tool for measuring and comparing phenomena that have a specific nature and property based on which changes in certain variables can be studied over a period [11].

In the general classification, theorizing strategies can be divided into three categories: Inductive strategies, deductive strategies, and reciprocal strategies (reproductive strategy), which are a combination of the two previous strategies. One of the inductive strategies is to theorize the ``axiomatic method with definitional reduction''. In this method, several variables are converted to a more general variable in a process; in other words, this method seeks to collapse several variables into one variable. Indexing should be considered as a tool and method in the service of this strategy [12].

In the simplest definition of an index, it can be considered ``a combination of two or more variables''. An index is a one-dimensional variable with some values that, when combined, eventually reach a single value. There may also be a two-dimensional variable or other types of indicators. An index is very similar to a multi-answer question or a list that is given to the respondent to choose the desired answer from it (inventory) and the only difference is that an index only covers one scope or a specific topic. To make an index, several questions combine the two answers and it is thought that such questions have some aspects in common [13].

To construct a good index, the question items must be related to each other and have a significant correlation. The index must be valid, i.e., what is expected to be measured by the index be measured accurately and reliably, and ultimately the name or title given to the index must not be misleading. The index should be such that maximum inference can be made from the pattern. It should be constant and not sensitive to any partial variable of the pattern. This principle means that the value of the variables used in the index may not change, but the index should still be relatively constant. Moreover, this index should be simple and allow comparison, be composed of several variables, and be able to interpret and analyze. It must also have a meaning beyond its mathematical definition [14].

In its simplest form, the researcher combines the variables after selecting the appropriate signs or reagents. In this combination, the researcher can calculate the relative weights of each material as well as the calculation methods and the range of their changes arbitrarily and mentally. The complex form of this work is the use of complex statistical techniques related to multivariate regression and factorial analysis. One of the simplest methods of indexing is to summarize the number of correct answers to the questions that are thought to be related to the concept in question [15]. The purpose of this article was to extract the data related to the index of economic sanctions against the Islamic Republic of Iran. One of the data needed in the current situation in Iran is the data that can determine the impact of economic sanctions on the Iranian economy. To have a wide range of opinions, the fuzzy logic method was chosen as a verbal-numerical interface for indexing economic sanctions against the Islamic Republic of Iran. First, according to the year of the embargo and the sanctioning country, the questions were developed in the form of a questionnaire. To extract data consistent with the realities of macroeconomics, five fuzzy options were provided to the individuals to gather their opinions in more detail. Table 1 shows the fuzzy propositions and their numerical equivalents. After that, according to the table related to the numerical equivalents of fuzzy propositions, the geometric average of the opinions of 15 experts on economic sanctions against Iran was collected and used as the preliminary data. In these questionnaires, individuals were asked to express their views on the impact of each of the sanctions imposed on Iran on the variables of GDP, inflation rate, and unemployment rate. The year was examined according to the sanctioning body, including the United States, the United Nations Security Council, and the European Union. Sanctions imposed on Iran since 1979 have affected various sectors of the economy. Therefore, the questionnaires were developed according to the failure points in the basic macroeconomic variables.

**Table 1:** Numerical equivalent of fuzzy propositions Fuzzy equivalent Verbal expression.

Verbal expression	Fuzzy equivalent		
	Low	Medium	High
Very low	1	1	**1**
Low	2	3	**4**
Medium	4	5	**6**
High	6	7	**8**
Very High	9	9	**9**

### Fuzzy logic

Fuzzy logic is an approach in computer science. Its main idea was first proposed by Professor Lotfizadeh in 1976. Fuzzy logic 0 and 1 are assumed to be one limit state of reality, and in the meantime, several manual states [16]. The fuzzy equivalents for verbal preferences can be seen in Table 1. The most widely used fuzzy number is its triangular type. This number has three values including LOW, MEDIUM, and HIGH which are shown according to Eq. (1). The membership of triangular fuzzy numbers is in the form of Eq. (2).

(1) A=(Low,Medium,High)

(2) $$\mu_{A^{∼}}\left(x\right)=\left\{ \begin{array}{c} \frac{x-L}{M-L}\;L\leq x<M\\ \frac{H-x}{H-M}\;M\leq x<H\\ 0\;x<L,x>H \end{array}\right.$$

The steps for fuzzy logic are as follows:

**Step (1):** In this step, verbal variables are considered as input and output. This data was collected through a questionnaire from 15 activists and experts in the field of economics of sanctions. Linguistic criteria were used in this method according to Table 1.
**Step (2):** In this step, it is necessary to define the appropriate membership function for fuzzy verbal data. In most studies, the triangular membership function is used. Therefore, in this article, to extract the data related to the sanction index, the economic triangular membership function was used.
**Step (3):** In this step, it is essential to determine the rules governing fuzzy logic. Therefore, appropriate rules must be applied by the researcher to the fuzzy system.
**Step (4):** Obtaining fuzzy values; in this step, according to the completed questionnaires, the input values were applied to the fuzzy system and were obtained for the output of fuzzy data.
**Step (5):** In this step, it is necessary to perform defuzzy operations to convert the fuzzy data to numerical data.

**Fig. 1:**
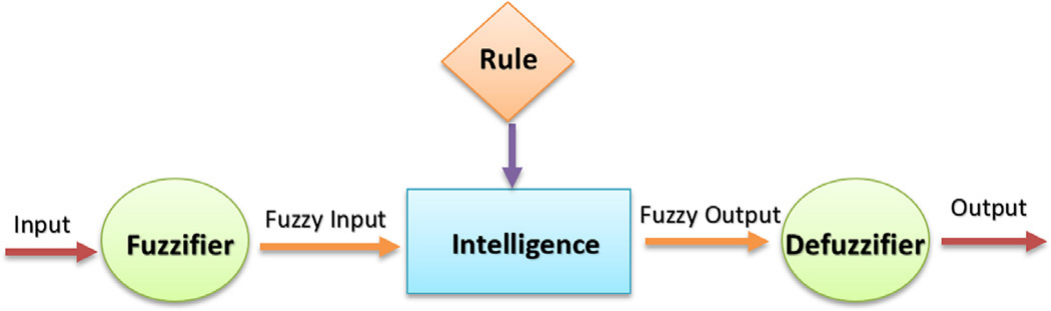
Fuzzy logic system.

In Fig. 1, the general structure of a fuzzy system and its components are shown.

The data used to extract the index of economic sanctions were obtained from fuzzy questionnaires. Using the years of economic sanctions against Iran and the failures in the three key macroeconomic variables, the questions were formulated and provided to 15 activists and experts in the economics of sanctions. In these questionnaires, the experts were asked about the impact of each of the economic sanctions on inflation, GDP, and unemployment rates in Iran. Five fuzzy reports were provided to the participants to broaden the range of their answers to these questions. The questionnaires were the result of aggregating the opinions of 15 activists and experts in the economics of sanctions on three important macroeconomic variables.

**Fig. 2:**
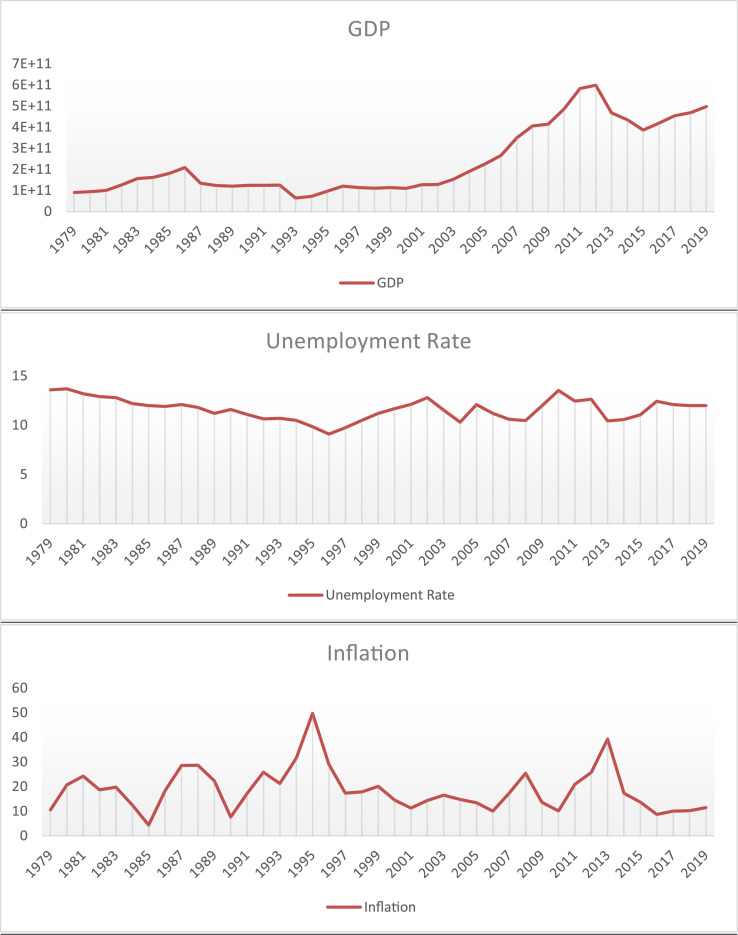
Graph of raw data related to GDP, the inflation rate, and the unemployment rate.

After collecting the questionnaires, each of the verbal propositions according to Table 1 was equated with a triangular fuzzy number. Then, a fuzzy system was designed according to three inputs and one output. Data presented in Fig. 2 shows the time series in the period of 1979–2019 for the variables GDP, unemployment rate, and inflation rate.

After collecting the questionnaires according to Table 1, the comments became fuzzy equivalents. Table 2 shows the values of the fuzzy input data of the questionnaires. These data are the result of the geometric average of the comments of all the activists and experts. Economics is in the context of the economics of sanctions.

**Table 2:** Fuzzy input data.

	GDP Input Data			Inflation Input Data			Unemployment Input Data		
	Low	Medium	High	Low	Medium	High	Low	Medium	High
**1**	1.51	1.85	2.15	5.38	6.36	7.32	1.31	1.43	1.52
**2**	3.60	4.66	5.70	1.66	2.15	2.58	1.14	1.19	1.23
**3**	6	7	8	6.16	7.11	8.06	1.09	1.15	1.20
**4**	1.44	1.79	2.09	1.51	1.93	2.29	1.25	1.38	1.48
**5**	6.68	7.44	8.16	1.90	2.47	2.99	1.74	2.31	2.83
**6**	5.83	6.80	7.75	6.33	7.23	8.12	1.20	1.34	1.44
**7**	5.68	6.69	7.69	8.07	8.41	8.72	1.04	1.07	1.09
**8**	2	2.66	3.28	2.51	3.55	4.57	6	6.92	7.82
**9**	1.04	1.07	1.09	4.03	5.05	6.06	5.18	6.01	6.83
**10**	1.31	1.55	1.74	6.50	7.27	8	2	3	4
**11**	8.07	8.36	8.62	6	6.96	7.90	7.65	8.04	8.39
**12**	7.44	8	8.51	6	7	8	8.75	8.85	8.92
**13**	7.65	8.04	8.39	8.52	8.70	8.85	6.50	7.36	8.19
**14**	6.68	7.48	8.25	9	9	9	6.16	7.11	8.06
**15**	4.45	5.46	6.47	7.44	8	8.51	4.70	5.72	6.73
**16**	5.68	6.69	7.69	6.16	7.11	8.06	8.29	8.55	8.79
**17**	6.68	7.48	8.25	6.50	7.36	8.19	6	7	8

**Fig. 3:**
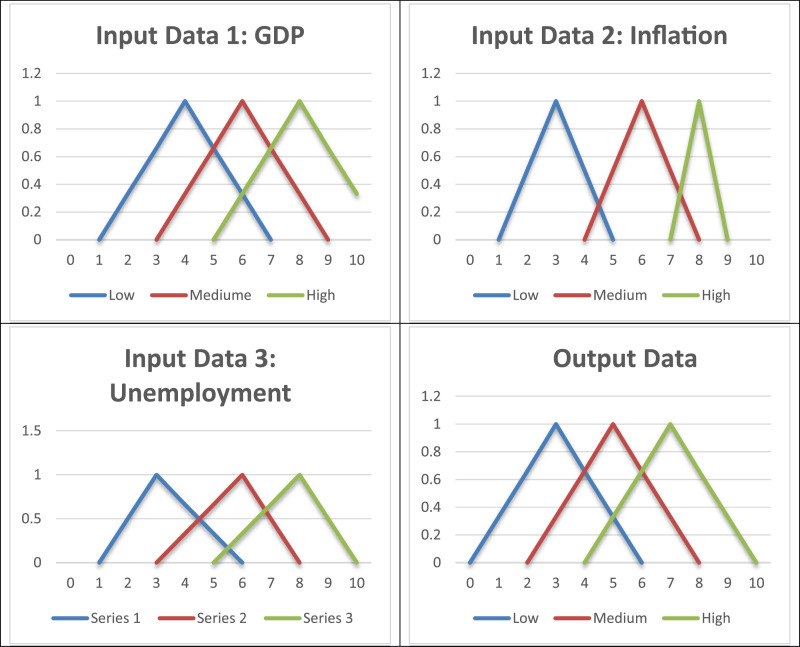
Triangular membership functions related to the inputs and outputs of fuzzy systems.

In this paper, to extract the sanction index, first a fuzzy logic system with three inputs and one output was designed. Fig. 3 shows the membership function for each component of this triangular fuzzy system.

The fuzzy input values were then designed into a three-input and one-output fuzzy system, and Table 3 shows the fuzzy output values of the economic sanction index designed from the fuzzy system.

**Table 3:** Output data from the fuzzy system.

	Output Data: Sanction Index			
	Low	Medium	High	Fuzzy Data
**1**	1.42	1.84	6.35	3.21
**2**	1.19	2.13	4.66	2.66
**3**	1.15	7	7.11	5.08
**4**	1.38	1.78	1.91	1.69
**5**	2.30	2.46	7.43	4.06
**6**	1.33	6.80	7.23	5.12
**7**	1.07	6.69	8.40	5.39
**8**	2.65	3.55	6.91	4.37
**9**	1.07	5.05	6	4.04
**10**	1.54	3	7.26	3.93
**11**	6.95	8.03	8.35	7.78
**12**	7	7.99	8.84	7.94
**13**	7.35	8.03	8.69	8.02
**14**	7.11	7.47	9	7.86
**15**	5.46	5.71	7.99	6.39
**16**	6.69	7.11	8.55	7.45
**17**	7	7.35	7.47	7.27

Table 4 shows the time series of the data related to Iran's economic sanction index.

**Table 4:** Sanction index data for Iran.

Sanction Index Data					
Year	Sanction Index	Year	Sanction Index	Year	Sanction Index
**1979**	3.211614	**1993**	4.331441	**2007**	5.860179
**1980**	2.9383	**1994**	4.595396	**2008**	7.784207
**1981**	2.664987	**1995**	5.123305	**2009**	7.865674
**1982**	3.271227	**1996**	5.391473	**2010**	7.947141
**1983**	3.877467	**1997**	4.374773	**2011**	7.988276
**1984**	5.089946	**1998**	4.292395	**2012**	8.029411
**1985**	4.241109	**1999**	4.210017	**2013**	7.864564
**1986**	3.392271	**2000**	4.127639	**2014**	6.393815
**1987**	1.694595	**2001**	4.045261	**2015**	6.658696
**1988**	1.991207	**2002**	4.017983	**2016**	6.923576
**1989**	2.287818	**2003**	3.990706	**2017**	7.188457
**1990**	2.881041	**2004**	3.977067	**2018**	7.453338
**1991**	3.474263	**2005**	3.963429	**2019**	7.277496
**1992**	4.067486	**2006**	3.936151		

## Results

In this article, the aim was to provide a suitable quantitative index for the study of economic sanctions against Iran. Given the above, it seems necessary to construct a suitable quantitative index to examine all the aspects of sanctions. For this purpose, the fuzzy logic method was chosen to extract the sanction index. This method, due to the inclusion of a wide range of verbal propositions, allows the researcher to quantify the studied qualitative phenomenon more accurately.

In this regard, a statistical population consisting of 15 experts who were active in the field of economics of sanctions and were graduates of doctoral and master's degrees in international economics was selected and the research questionnaires were provided to them. In these questionnaires, the experts were asked to use fuzzy verbal interfaces to assess the impact of each of the imposed sanctions on GDP, the inflation rate, and the unemployment rate in the Iranian economy. The variables GDP, inflation rate, and unemployment rate were selected because economic sanctions have a direct effect on them. They have been affected by economic sanctions during the period of 1979–2019. Therefore, it can be said that this index is based on GDP, inflation, and unemployment rates in Iran.

According to the results of the fuzzy logic output, the impact of sanctions on Iran's economy can be seen in the form of a time series. The results showed that the sanctions imposed on the Islamic Republic of Iran in 2012 had the most negative economic effects on the Iranian economy. After that, according to the values obtained for the sanction index, the sanctions imposed in 2011 were able to greatly affect the Iranian economy. This trend indicates that over time, the sanctions against the Islamic Republic of Iran have had more severe effects and consequences. Therefore, it is necessary to place serious measures in the field of self-sufficiency, self-reliance, reduction of dependence on oil, proper selection of strategic partners, and such issues on the agenda of international economic activists in order to be aware of the effects and consequences of sanctions and minimize them, thus making the country resistant to these oppressive acts.

## Conclusion

The aim of this article was to provide a suitable quantitative index for the study of economic sanctions against Iran. Given the above, it seems necessary to construct a suitable quantitative index to examine all the aspects of sanctions. For this purpose, the fuzzy logic method was chosen to extract the sanction index. This method, due to the inclusion of a wide range of verbal propositions, allows the researcher to quantify the studied qualitative phenomenon more accurately.

In this regard, a statistical population consisting of 15 experts who were active in the field of sanction economics and were Ph.D. graduates in international economics was selected and research questionnaires were provided to them. In these questionnaires, the experts were asked to use the interface fuzzy verbal assessments of the impact of each of the imposed sanctions on GDP, the inflation rate, and the unemployment rate in the Iranian economy. The variables of GDP, the inflation rate, and the unemployment rate were selected because economic sanctions have a direct effect on them and during the period of 1979–2019, they were affected by the economic sanctions. Therefore, it can be said that this index is based on production, inflation, and employment in Iran.

According to the results of the fuzzy logic output, the impact of sanctions on Iran's economy can be seen in the form of a time series. The results showed that the sanctions imposed on the Islamic Republic of Iran in 2012 had the most negative effects on the Iranian economy. After that, according to the values obtained for the sanction index, the sanctions imposed in 2011 were able to greatly affect the Iranian economy. This trend indicates that over time, the sanctions against the Islamic Republic of Iran have had more severe effects and consequences. Therefore, it is necessary to place serious measures in the field of self-sufficiency, self-reliance, reduction of dependence on oil, proper selection of strategic partners, and such issues on the agenda of international economic activists to be aware of the effects and consequences of sanctions and minimize them, thus making the country resistant to these oppressive acts.

## Direct submission or co-submission

Direct Submission

## Declaration of Competing Interest

The authors declare that they have no known competing financial interests or personal relationships that could have appeared to influence the work reported in this paper.

## Appendix Supplementary materials

Image, application 1

## References

[1] Torbat A.E. (2020). The economic sanctions against Iran. Politics of Oil and Nuclear Technology in Iran.

[2] Gurvich E., Prilepskiy I. (2016). The impact of financial sanctions on the Russian economy. Voprosy Economiki.

[3] Ahmadreza Z. (2020). Some economic ideas in sanction times in history of Iran. Proceedings of the Міжнародне науково-технічне співробітництво: принципи, механізми, ефективність.

[4] Dong Y., Li C. (2018). Economic sanction games among the US, the EU and Russia: payoffs and potential effects. Econ. Model..

[5] Raman B.V., Bouwmeester R., Mohan S. (2009). Fuzzy logic water quality index and importance of water quality parameters. Air Soil Water Res..

[6] Gentile M., Rogers W.J., Mannan M.S. (2003). Development of a fuzzy logic-based inherent safety index. Process Safety Environ. Protect..

[7] Hendiani S., Bagherpour M. (2019). Developing an integrated index to assess social sustainability in construction industry using fuzzy logic. J. Cleaner Product..

[8] Shahrestani H., Anaraki N.K. (2008). How would a possible UN sanction affect the Iranian economy?. Glob. J. Bus. Res..

[9] Ismail A., Amjad S. (2014). Determinants of terrorism in Pakistan: an empirical investigation. Econ. Model..

[10] Shearkhani Sara, Mohammadi Teimour, Hadinejad Manijeh (2010). Examine sanctions efficiency against Iran's non-oil. Trade.

[11] Hanai T., Ushijima K., Tanaka T., Aoki H., Tomoda A. (2020). Index Tree Search Method and Computer.

[12] Sokolov E.L., Chechik A.A., Elokhovskiy V.Y., Kolonitsky D.I. (2019). Method for Measuring Glycemic Index of Human-Consumed Food.

[13] Liu S., Xu X., Chuang T.D., Sun Y.C., Wang-Lin L., Huang Y.W., Ye J. (2019). Method and Apparatus for Palette index Coding in Video and Image Compression.

[14] Kiyoung H. (2020). Method for Reassigning Root Sequence Index and Apparatus Therefore.

[15] Räsänen E., Kikta R., Sorvari A., Salmenkaita J.P., Huhtala Y., Mannila H., Murto J. (2018). Location-Based Novelty Index Value and Recommendation System and Method.

[16] L.A. Zadeh, (1776). Toward human level machine intelligence—is it achievable? The Need for a Paradigm Shift. U

